# Transcriptional activity of vitamin D receptor in human periodontal ligament cells is diminished under inflammatory conditions

**DOI:** 10.1002/JPER.19-0541

**Published:** 2020-06-21

**Authors:** Alice Blufstein, Christian Behm, Barbara Kubin, Johannes Gahn, Andreas Moritz, Xiaohui Rausch‐Fan, Oleh Andrukhov

**Affiliations:** ^1^ Department of Conservative Dentistry and Periodontology University Clinic of Dentistry Medical University of Vienna Vienna Austria

**Keywords:** inflammation, mesenchymal stem cells, periodontal ligament, VDR, vitamin D

## Abstract

**Background:**

Although vitamin D_3_ deficiency is considered as a risk factor for periodontitis, supplementation during periodontal treatment has not been shown to be beneficial to date. Human periodontal ligament cells (hPDLCs) are regulated by vitamin D_3_ and play a fundamental role in periodontal tissue homeostasis and inflammatory response in periodontitis. The aim of this study is to investigate possible alterations of the vitamin D_3_ activity in hPDLCs under inflammatory conditions.

**Methods:**

Cells isolated from six different donors were treated with either 1,25(OH)_2_D_3_ (0 to 10 nM) or 25(OH)D_3_ (0 to 100 nM) in the presence and absence of ultrapure or standard *Porphyromonas gingivalis* lipopolysaccharide (*Pg*LPS), Pam3CSK4, or interferon‐γ for 48 hours. Additionally, nuclear factor (NF)‐κB inhibition was performed with BAY 11‐7082. The bioactivity of vitamin D in hPDLCs was assessed based on the gene expression levels of vitamin D receptor (VDR)‐regulated genes osteocalcin and osteopontin. Additionally, VDR and CYP27B1 expression levels were measured.

**Results:**

The vitamin D_3_‐induced increase of osteocalcin and osteopontin expression was significantly decreased in the presence of standard *Pg*LPS and Pam3CSK4, which was not observed by ultrapure *Pg*LPS. Interferon‐y had diverse effects on the response of hPDLCs to vitamin D_3_ metabolites. NF‐kB inhibition abolished the effects of standard *Pg*LPS and Pam3CSK4. Standard *Pg*LPS and Pam3CSK4 increased VDR expression in the presence of vitamin D_3_. CYP27B1 expression was not affected by vitamin D_3_ and inflammatory conditions.

**Conclusions:**

This study indicates that the transcriptional activity of VDR is diminished under inflammatory conditions, which might mitigate the effectiveness of vitamin D_3_ supplementation during periodontal treatment.

## 1 INTRODUCTION

The secosteroid hormone vitamin D_3_ is either produced by the skin under the influence of ultraviolet light of the sun or obtained as a dietary supplement.^1^ Following hydroxylation into 25‐hydroxyvitamin D_3_ (25(OH)D₃) in the liver, it is further converted in the kidney by the 1α‐hydroxylase (CYP27B1) into the most potent metabolite 1,25‐dihydroxyvitamin D_3_ (1,25(OH)₂D₃).^2^ The functions of CYP27B1 are opposed by 24‐hydroxylase (CYP24A1), which inactivates 1,25(OH)₂D₃.^3^ Although kidney and liver are considered to be the main sites involved in vitamin D₃ metabolism, local conversion, and inactivation in various cell types and tissues has been reported.^4^, ^5^, ^6^ Binding of 1,25(OH)₂D₃ to the vitamin D receptor (VDR) leads to expression of genes responsible for mediating the effects of vitamin D_3_.^7^ In particular, 1,25(OH)₂D₃ plays a significant role for bone metabolism by regulating calcium and phosphate concentration.^8^ Further, immunomodulatory, anti‐inflammatory, and anti‐proliferative effects have been reported.^9^, ^10^

Due to its various positive effects, vitamin D_3_ supplementation has been targeted as a potential adjuvant therapeutic approach for many different inflammatory diseases, including periodontitis.^9^, ^11^ This chronic inflammatory disease leads to destruction of tooth supporting tissues including the alveolar bone.^12^ Periodontitis is associated with a dysbiosis within the host‐microbial interaction and is influenced by various risk factors.^13^, ^14^ To date, data concerning association of vitamin D_3_ deficiency with periodontitis and the effects of vitamin D_3_ supplementation on the treatment outcome are controversial.^15^ While some cross‐sectional observational studies showed associations between vitamin D_3_ deficiency and periodontitis risk, there are longitudinal studies claiming the opposite.^15^, ^16^, ^17^ Furthermore, only few studies observed a positive impact of vitamin D_3_ supplementation on the outcome of periodontal surgical procedures or on clinical parameters during the maintenance phase.^18^, ^19^ To the best of our knowledge, there is no evidence of beneficial effects of vitamin D_3_ supplementation during the initial non‐surgical treatment phase.

Human periodontal ligament cells (hPDLCs) play an integral part in maintaining periodontal tissue homeostasis.^20^ They possess mesenchymal stem cell (MSC) character, exhibiting characteristic MSC surface markers and the ability to differentiate in vitro into various cell types including osteoblasts.^21^, ^22^ Several studies have revealed the effects of vitamin D₃ on hPDLCs. For example, these cells express VDR and are able to convert 25(OH)D₃ into 1,25(OH)₂D₃ via CYP27B1. Additionally, 1,25(OH)₂D₃ enhances osteogenic and immunomodulatory activity of hPDLCs. Furthermore, both vitamin D_3_ metabolites decrease the inflammatory response of hPDLCs to bacterial components.^5^, ^23^, ^24^, ^25^

Given the fact that vitamin D_3_ has such beneficial effects on bone metabolism and resolution of inflammation, there is no reasonable explanation for the divergence in the existing data concerning vitamin D_3_ as a risk factor for periodontitis and supplementation during periodontal treatment. As hPDLCs have been shown to exhibit compromised functions under inflammatory conditions, this may be a potential factor altering the activity of vitamin D_3_ metabolites, but such a possibility was never studied to date.^26^

This study aims to investigate the bioactivity of 1,25(OH)₂D₃ and 25(OH)D₃ in hPDLCs under inflammatory conditions. The bioactivity of vitamin D_3_ metabolites was assessed based on the expression of VDR regulated genes such as bone γ‐carboxyglutamic acid‐containing protein (BGLAP or osteocalcin) and secreted phosphoprotein 1 (SPP1 or osteopontin) on both gene and protein levels. In addition, the gene expression levels of vitamin D_3_‐related molecules VDR and CYP27B1 were assessed under the same treatment modalities. Inflammatory conditions were simulated by stimulation with lipopolysaccharide (LPS) of the periodontopathogenic bacterium *Porphyromonas gingivalis (Pg)* (standard and ultrapure preparations), synthetic Toll‐like receptor (TLR)‐2 agonist Pam3CSK4, or proinflammatory cytokine interferon (IFN)‐γ. To assess the role of nuclear factor (NF)‐κB activation, experiments were additionally performed with NF‐κB inhibitor BAY 11‐7082. We hypothesized that the response of hPDLCs to vitamin D_3_ metabolites and the expression of VDR may be altered under inflammatory conditions.

## 2 MATERIALS AND METHODS

### 2.1 Cell culture

The protocol for isolation of primary hPDLCs applied in this study was approved by the ethics committee of the Medical University of Vienna (ethical approval number: 1694/2015, revised in 2018). Experiments were performed following the “Good Scientific Practice” guidelines of the Medical University of Vienna and the Declaration of Helsinki.

Periodontal ligament tissue was obtained from third molars of six periodontally healthy individuals (three females, three males) undergoing tooth extraction as part of their orthodontic treatment plan. The donors, which were white non‐smokers aged 18 and 24 years, had no systemic or oral diseases. They were informed in detail about the study and gave their written consent before the procedure. Primary hPDLCs isolation and expansion was executed as reported in our previous study.^27^ Subsequently, hPDLCs were cultivated in Dulbeccos modified Eagle´s medium (DMEM),* supplemented with 10% fetal bovine serum (FBS)† and 1% penicillin and streptomycin (P/S),‡ under humidified conditions at 37°C. Confirmation of MSC character was performed with flow cytometry, analyzing MSC surface markers (CD29, CD90, CD105, CD146)§ and hematopoietic cell surface markers (CD14, CD31, CD34, CD45).¶

### 2.2 Treatment protocol

hPDLCs within passage three to six were seeded in 24‐well plates at a density of 5 × 10^4^ in 0.5‐mL DMEM supplemented with 1% P/S and 10% FBS and incubated overnight. Stimulation was performed with either 1,25(OH)_2_D_3_ (0.1 to 10 nM)# or 25(OH)D_3_ (10 to 100 nM)∥ in FBS‐free medium containing 1% P/S.^25^, ^28^, ^29^, ^30^, ^31^ Inflammatory conditions were simulated with pro‐inflammatory mediators *Pg*LPS (standard and ultrapure, 1 µg/mL)**, synthetic TLR‐2 agonist Pam3CSK4 (1 µg/m)†† or IFN‐γ (0.1 µg/mL).‡‡ Treatment with standard and ultrapure *Pg*LPS was performed in combination with soluble CD14 (sCD14; 0.2 µg/mL),§§ because it enhances the reaction of hPDLCs to LPS due to a lack of membrane bound CD14.^32^ In addition, NF‐κB inhibition was performed with BAY 11‐7082¶¶ (0.3 µg/mL) in hPDLCs treated with 10 nM 1,25(OH)_2_D_3_ or 100 nM 25(OH)D_3_ in the presence and absence of standard *Pg*LPS and Pam3CSK4. Untreated hPDLCs were used as control group. Experiments were performed in duplicates for every donor.

### 2.3 Quantitative polymerase chain reaction

Gene expression analysis of BGLAP, SPP1, and VDR was performed with quantitative polymerase chain reaction (qPCR). All steps were performed with commercially available kits## according to the manufacturer's instructions, namely cell lysis, mRNA extraction, transcription into cDNA, and qPCR. In the following, reverse transcription was performed with a thermocycler.∥∥ The ABI StepOnePlus device was used for qPCR, using following gene expression assays***: BGLAP, Hs01587814_g1; SPP1, Hs00959010_m1; VDR, Hs_00172113_m1; CYP27B1, Hs_00168017_m1. The analysis was performed in duplicates with following thermocycler††† conditions: 95°C for 10 minutes; 40 cycles, each for 15 seconds at 95°C; 60°C for 1 minute. To evaluate the mRNA expression levels of BGLAP, SPP1, VDR, and CYP27B1, the cycle threshold (C_t_) for each sample was determined and changes in the target gene expression were calculated with the 2^−∆∆Ct^ method: ΔΔC_t_   =   (C_t_ ^target^−C_t_ ^GAPDH^)_sample_−(C_t_ ^target^−C_t_ ^GAPDH^)_control_. Untreated hPDLCs served as control group and GAPDH was used as endogenous control.

### 2.4 Enzyme‐linked immunosorbent assay

Osteocalcin and osteopontin protein concentrations in the cell culture supernatants were analyzed with enzyme‐linked immunosorbent assay (ELISA). For this purpose, commercially available kits‡‡‡ with a sensitivity of 156.5 pg/mL for osteocalcin and 31.25 pg/mL for osteopontin were used according to the manufacturer´s instructions. Samples were applied in duplicates and measurement of optical densities was performed with a photometer at 450 nm. Subsequently, they were plotted against a standard curve to determine the protein concentrations.

### 2.5 Statistical analysis

Mean values of six different donors were used for statistical analysis, which was executed with SPSS 24.0.§§§ Statistical differences between groups were analyzed by ANOVA for repeated measures, followed by post‐hoc paired *t*‐test. *P*‐values <0.05 were considered as statistically significant. Data are presented as mean values ± SEM.

## 3 RESULTS

### 3.1 Effect of vitamin D_3_ metabolites on the gene expression of osteocalcin by hPDLCs under physiological and inflammatory conditions

**FIGURE 1:**
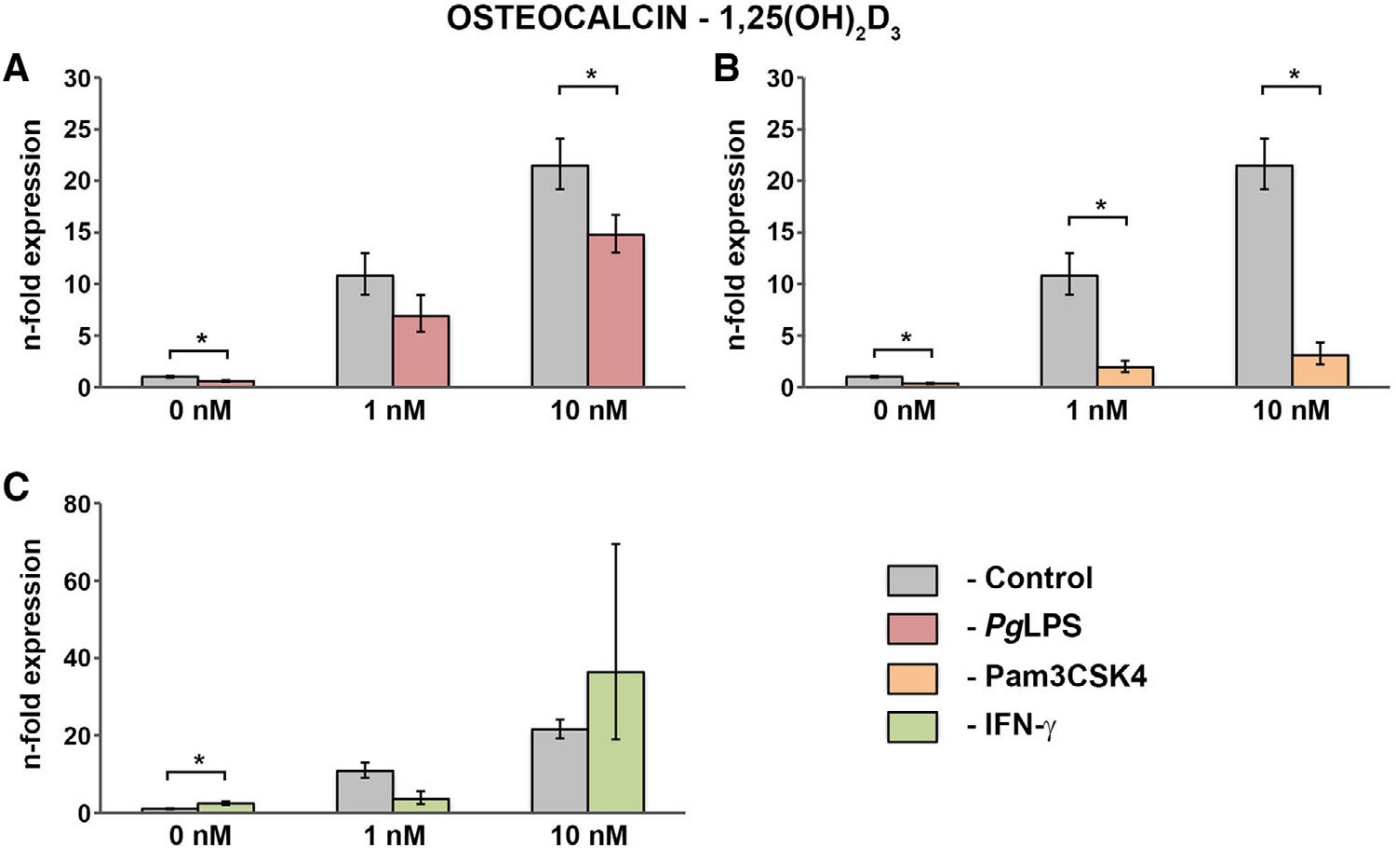
Gene expression of osteocalcin in hPDLCs treated with 1,25(OH)_2_D_3_ under physiological and inflammatory conditions hPDLCs of six healthy donors were treated with 1,25(OH)_2_D_3_ (1 nM, 10 nM) in the presence and absence of standard *Pg*LPS (1 µg/mL; **A**) + sCD14 (0.2 µg/mL), Pam3CSK4 (1 µg/mL; **B**) or IFN‐γ (0.1 µg/mL; **C**) for 48 hours. Osteocalcin gene expression levels were measured with qPCR. Y‐axes show the n‐fold expression of osteocalcin expression compared with untreated cells ( = 1). GAPDH served as endogenous control. Data are presented as mean ± SEM of six different donors. *Significant difference between groups, *P* <0.05

The gene expression levels of osteocalcin induced by 1,25(OH)_2_D_3_ in concentrations of 0 nM, 1 nM, and 10 nM alone or in combination with either standard *Pg*LPS(1 µg/mL; Figure 1A), Pam3CSK4 (1 µg/mL; Figure 1B) or IFN‐γ (0.1 µg/mL; Figure 1C) are presented in Figure 1. 1,25(OH)_2_D_3_ led to a concentration‐dependent enhancement of osteocalcin under both physiological and inflammatory conditions. Treatment with Pam3CSK4 resulted in a significantly lower osteocalcin expression induced by all tested 1,25(OH)_2_D_3_ concentrations compared with their respective control groups. In detail, Pam3CSK4 led to a 5‐fold decrease of the 1 nM‐triggered gene expression of osteocalcin and a 7‐fold decrease of the 10 nM‐triggered osteocalcin expression. Similarly, stimulation with standard *Pg*LPS significantly diminished the effect of 10 nM 1,25(OH)_2_D_3_. Combined stimulation of hPDLCs with IFN‐γ and 1 nM 1,25(OH)_2_D_3_ resulted in a lower osteocalcin expression compared with the same 1,25(OH) _2_D_3_ concentration alone. Contrary, IFN‐γ increased the effects of 10 nM 1,25(OH)_2_D_3_ on the osteocalcin expression by about 1,7‐fold in comparison with separate stimulation with the vitamin D_3_ metabolite. However, the effects of IFN‐γ had no statistical significance.

**FIGURE 2:**
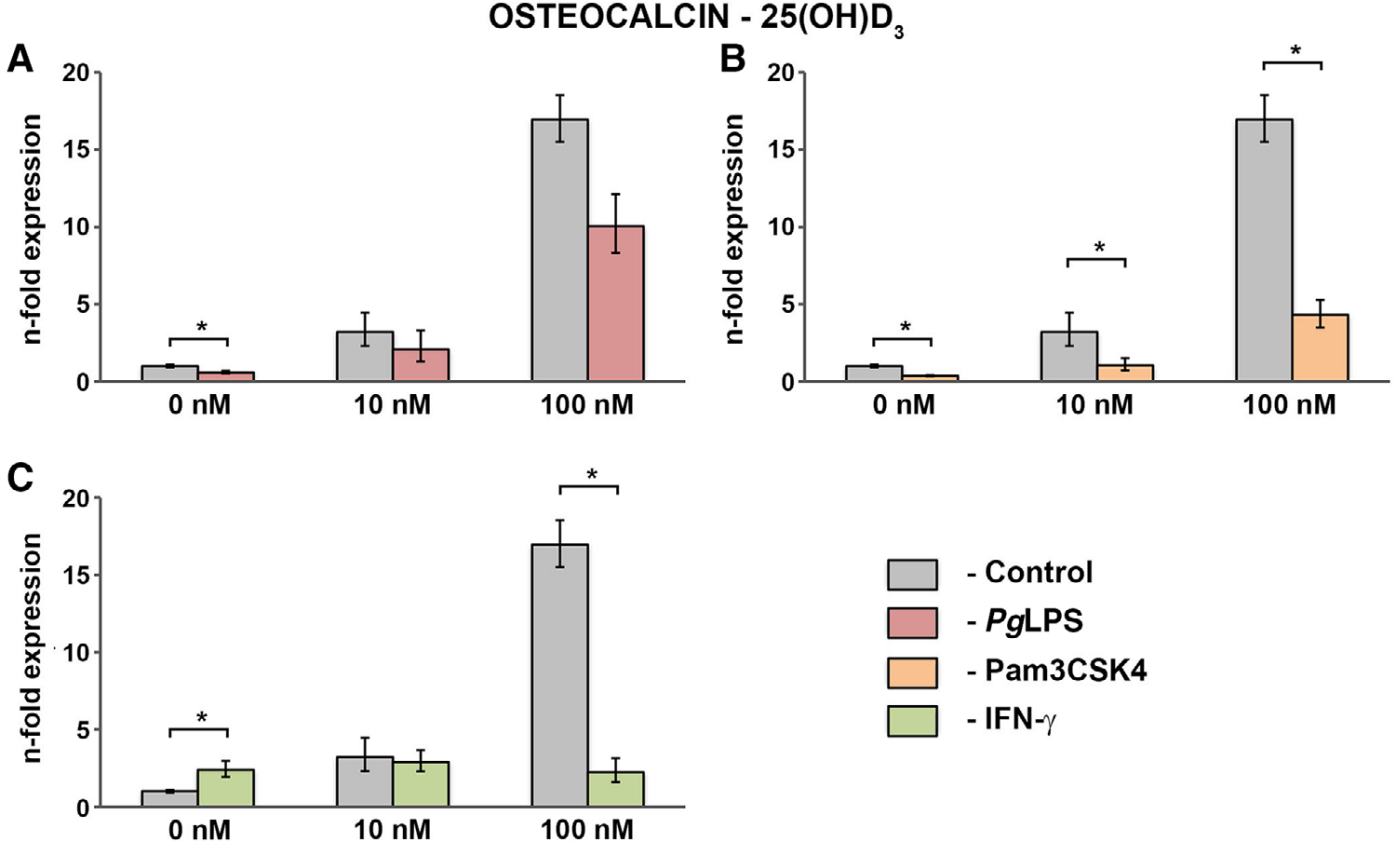
Gene expression of osteocalcin in hPDLCs treated with 25(OH)D_3_ under physiological and inflammatory conditions hPDLCs of six healthy donors were treated with 25(OH)D3 (10 nM, 100 nM) in the presence and absence of standard *Pg*LPS (1 µg/mL; **A**) + sCD14 (0.2 µg/mL), Pam3CSK4 (1 µg/mL; **B**) or IFN‐γ (0.1 µg/mL; **C**) for 48 hours. Osteocalcin gene expression levels were measured with qPCR. Y‐axes show the n‐fold expression of osteocalcin expression compared with untreated cells ( = 1). GAPDH served as endogenous control. Data are presented as mean ± SEM of six different donors. *Significant difference between groups, *P* <0.05

Figure 2 shows the response of hPDLCs to various concentrations of 25(OH)D_3_ (0 to 100 nM) in the presence and absence of standard *Pg*LPS (1 µg/mL; Figure 2A), Pam3CSK4 (1 µg/mL; Figure 2B), and IFN‐γ (0.1 µg/mL; Figure 2C). Co‐stimulation of Pam3CSK4 with 10 nM 25(OH)D_3_ significantly reduced osteocalcin gene expression levels compared with physiological conditions by about three times. Likewise, the effects of the highest 25(OH)D_3_ concentration significantly decreased under the influence of Pam3CSK3 by about four times. Similar tendencies were observed for hPDLCs treated with standard *Pg*LPS and 25(OH)D_3_, but these effects were not statistically significant. Treatment with IFN‐γ did not affect the 10 nM 25(OH)D_3_‐triggered enhancement of the osteocalcin gene expression, whereas the effects of 100 nM were significantly decreased by about eight times.

The effect of 1,25(OH)_2_D_3_ (0 to 10 nM) and 25(OH)D_3_ (0 to 100 nM) in the presence and absence of ultrapure *Pg*LPS (1 µg/mL) on the expression of osteocalcin in hPDLCS is presented in supplementary Figure S1 in online *Journal of Periodontology*. Stimulation with ultrapure *Pg*LPS did not significantly affect the response of hPDLCs to 1,25(OH)_2_D_3_ and 25(OH)D_3_.

### 3.2 Effect of vitamin D_3_ metabolites on the gene expression of osteopontin by hPDLCs under physiological and inflammatory conditions

**FIGURE 3:**
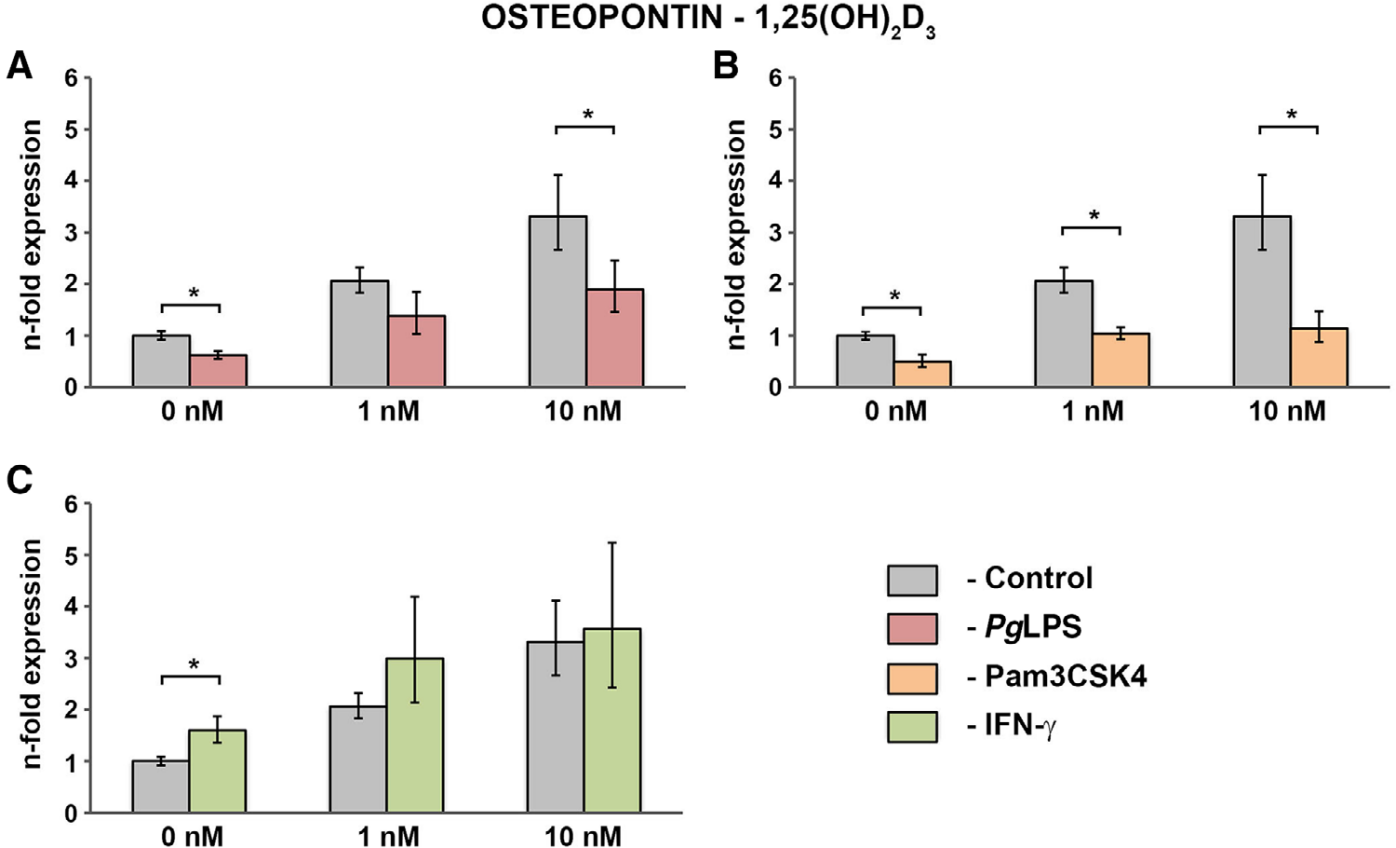
Gene expression of osteopontin in hPDLCs treated with 1,25(OH)_2_D_3_ under physiological and inflammatory conditions. hPDLCs of six healthy donors were treated with 1,25(OH)2D3 (1 nM, 10 nM) in the presence and absence of standard *Pg*LPS (1 µg/mL; **A**) + sCD14 (0.2 µg/mL), Pam3CSK4 (1 µg/mL; **B**) or IFN‐γ (0.1 µg/mL; **C**) for 48 hours. Osteopontin gene expression levels were measured with qPCR. Y‐axes show the n‐fold expression of osteopontin expression compared with untreated cells ( = 1). GAPDH served as internal reference. Data are presented as mean ± SEM of six different donors. *Significant difference between groups, *P* <0.05

The influence of different concentrations of 1,25(OH)_2_D_3_ (0 to 10 nM) alone or in co‐stimulation with either standard *Pg*LPS (1 µg/mL; Figure 3A), Pam3CSK4 (1 µg/mL; Figure 3B) or IFN‐γ (0.1 µg/mL; Figure 3C) on the gene expression levels of osteopontin are demonstrated in Figure 3. The osteopontin enhancement induced by 10 nM 1,25(OH)_2_D_3_ was significantly decreased in the presence of standard *Pg*LPS. Treatment with Pam3CSK4 similarly decreased the effect of 1 and 10 nM 1,25(OH)_2_D_3_ on osteopontin gene expression by >50% compared with physiological conditions. In contrast, IFN‐γ enhanced the effects of both 1 nM and 10 nM 1,25(OH)_2_D_3_ compared with separate treatment with the respective concentration of the vitamin D_3_ metabolite. However, these observations were not statistically significant.

**FIGURE 4:**
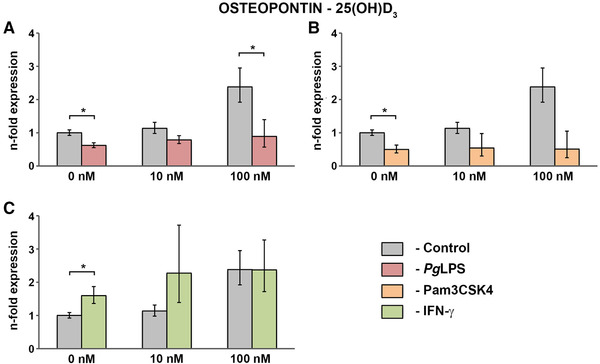
Gene expression of osteopontin in hPDLCs treated with 25(OH)D_3_ under physiological and inflammatory conditions. hPDLCs of six healthy donors were treated with 25(OH)D3 (10 nM, 100 nM) in the presence and absence of standard *Pg*LPS (1 µg/mL; **A**) + sCD14 (0.2 µg/mL), Pam3CSK4 (1 µg/mL; **B**), or IFN‐γ (0.1 µg/mL; **C**) for 48 hours. Osteopontin gene expression levels were measured with qPCR. Y‐axes show the n‐fold expression of osteopontin expression compared with untreated cells ( = 1). GAPDH served as endogenous control. Data are presented as mean ± SEM of six different donors. *Significant difference between groups, *P* <0.05

Resulting gene expression levels of osteopontin in hPDLCs treated with 25(OH)D_3_ in concentrations of 0 nM, 10 nM, and 100 nM alone and in combination with standard *Pg*LPS (1 µg/mL; Figure 4A), Pam3CSK4 (1 µg/mL; Figure 4B) or IFN‐γ (0.1 µg/mL; Figure 4C) are presented in Figure 4. The effects of 25(OH)D_3_ on the osteopontin expression were reduced in the presence of Pam3CSK4, but these differences were not statistically significant. Similar observations were made for co‐stimulation of 25(OH)D_3_ with standard *Pg*LPS compared with the vitamin D_3_ metabolite alone, which was significant in a concentration of 100 nM. Stimulation with IFN‐γ had no significant effect on the 25(OH)D_3_‐induced osteopontin gene expression levels in hPDLCs.

Gene expression levels of osteopontin after stimulation of hPDLCs with 0 to 10 nM 1,25(OH)_2_D_3_ or 0 to 100 nM 25(OH)D_3_ in the presence and absence of 1 µg/mL ultrapure *Pg*LPS is shown in supplementary Figure S1 in online *Journal of Periodontology*. Treatment of hPDLCs with ultrapure *Pg*LPS had no significant effect on the vitamin D_3_‐induced expression of osteopontin.

### 3.3 Effect of NF‐κB inhibition on vitamin D_3_‐induced gene expression of osteocalcin and osteopontin by hPDLCs under physiological and inflammatory conditions

**FIGURE 5:**
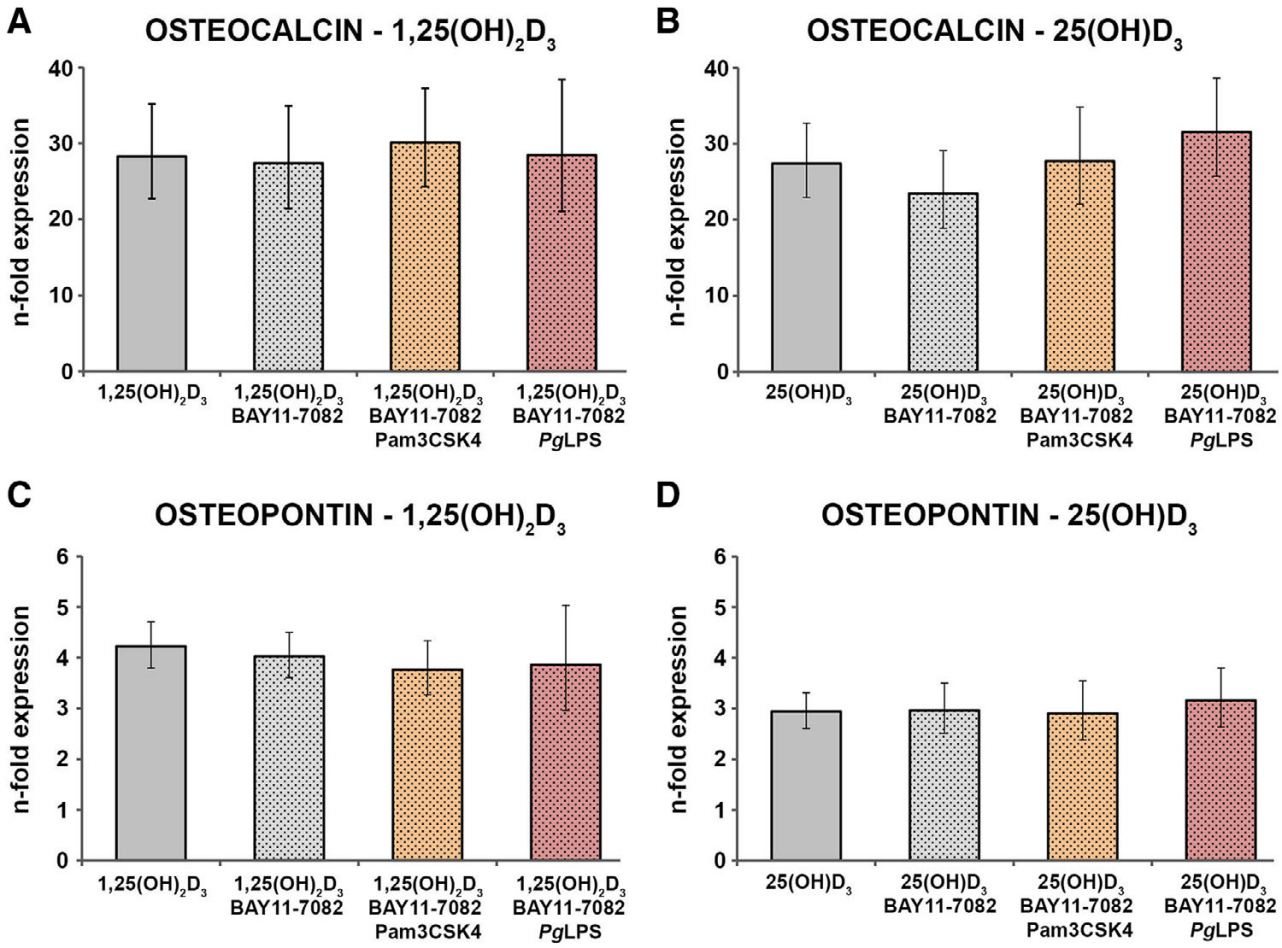
Gene expression levels of osteocalcin and osteopontin in hPDLCs treated with 1,25(OH)_2_D_3_ or 25(OH)D_3_ under physiological and inflammatory conditions and NF‐κB inhibition. hPDLCs of six healthy donors were treated with 1,25(OH)_2_D_3_ (10 nM; **A** and **C**) or 25(OH)D_3_ (100 nM; **B** and **D**) in the presence and absence of standard *Pg*LPS (1 µg/mL) + sCD14 (0.2 µg/mL) or Pam3CSK4 (1 µg/mL;) for 48 hours. In addition, experiments were performed in the presence and absence of NF‐κB inhibitor BAY 11‐7082 (0.3 µg/mL). Osteocalcin (A and B) and osteopontin (C and D) gene expression levels were measured with qPCR. Y‐axes show the n‐fold expression of osteocalcin and osteopontin expression, respectively, compared with untreated cells ( = 1). GAPDH served as endogenous control. Data are presented as mean ± SEM of six different donors

Resulting gene expression levels of osteocalcin in response to 1,25(OH)_2_D_3_ (10 nM; Figure 5A) or 25(OH)D_3_ (100 nM; Figure 5B) under physiological and inflammatory (1 µg/mL standard *Pg*LPS or 1 µg/mL Pam3CSK4) conditions in the presence and absence of NF‐κB inhibitor BAY 11‐7082 (0.3 µg/mL) are presented in Figure 5. The reduction of the vitamin D_3_‐induced osteocalcin expression by Pam3CSK4 and standard *Pg*LPS was significantly recovered in the presence of the NF‐κB inhibitor.

The effects of NF‐κB inhibitor BAY 11‐7082 on the 1,25(OH)_2_D_3_ (10 nM; Figure 5C) and 25(OH)D_3_ (100 nM; Figure 5D) triggered osteopontin expression in the presence and absence of standard *Pg*LPS (1 µg/mL) or Pam3CSK4 (1 µg/mL) is demonstrated in Figure 5. Similarly to osteocalcin, the decrease of the vitamin D_3_‐induced osteopontin expression was significantly recovered by inhibition of NF‐κB.

### 3.4 Effect of vitamin D_3_ metabolites on the gene expression of vitamin D_3_‐related molecules by hPDLCs under physiological and inflammatory conditions

**FIGURE 6:**
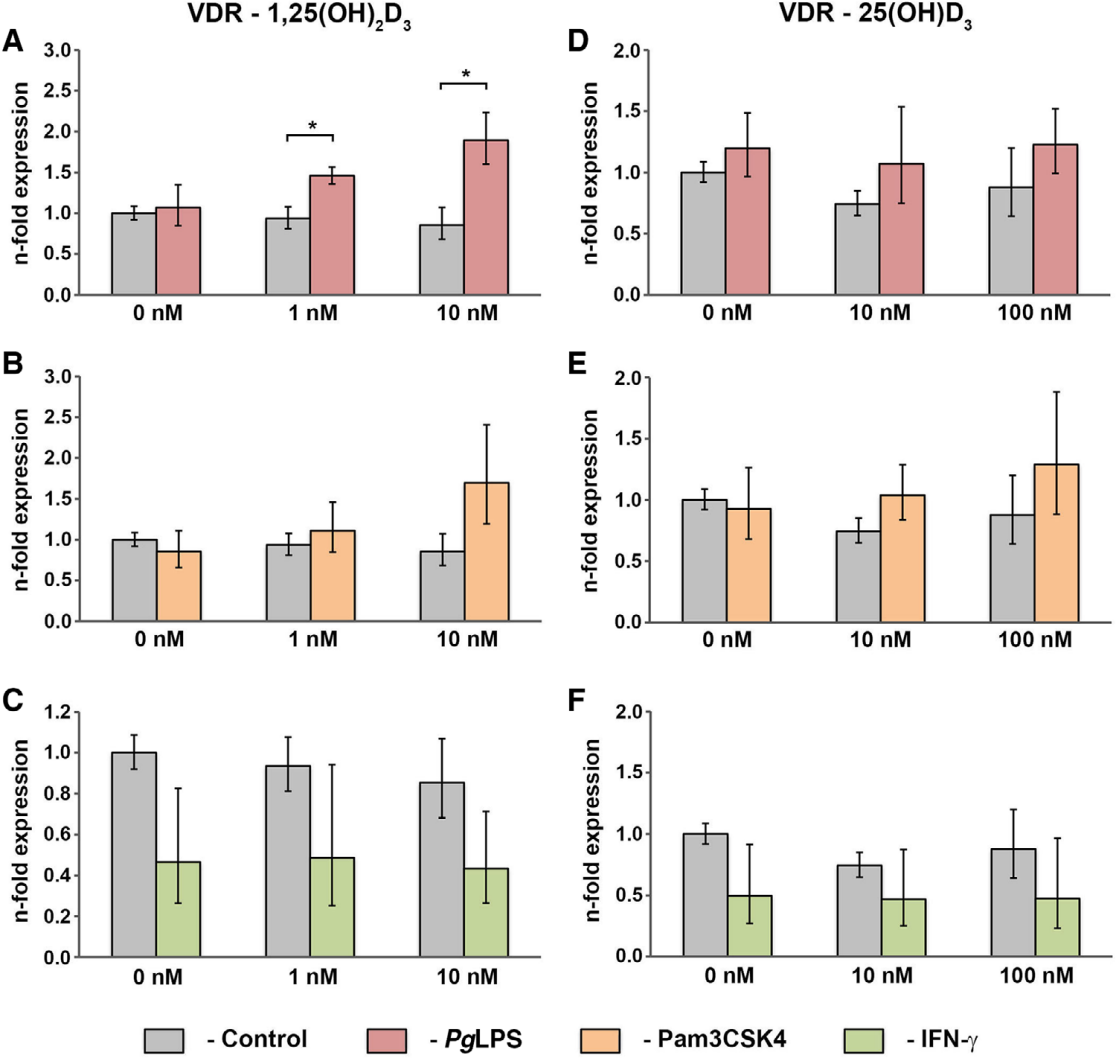
Gene expression of VDR in hPDLCs treated with 1,25(OH)_2_D_3_ or 25(OH)D_3_ under physiological and inflammatory conditions. hPDLCs of six healthy donors were treated with 1,25(OH)_2_D_3_ (1 nM, 10 nM; **A through C**) or 25(OH)D_3_ (10 nM, 100 nM; **D through F**) in the presence and absence of standard *Pg*LPS (1 µg/mL; A and D) + sCD14 (0.2 µg/mL), Pam3CSK4 (1 µg/mL; B and E), or IFN‐γ (0.1 µg/mL; C and F) for 48 hours. VDR gene expression levels were measured with qPCR. Y‐axes show the n‐fold expression of VDR expression compared with untreated cells ( = 1). GAPDH served as endogenous control. Data are presented as mean ± SEM of six different donors. *Significant difference between groups, *P* <0.05

Figure 6 shows the gene expression levels of VDR resulting from stimulation with 1,25(OH)_2_D_3_ (0 to 10 nM) in the presence and absence of standard *Pg*LPS (1 µg/mL; Figure 6A), Pam3CSK4 (1 µg/mL; Figure 6B), or IFN‐γ (0.1 µg/mL; Figure 6C). Under physiological conditions, 1,25(OH)_2_D_3_ tended to decrease gene expression levels of VDR, but not statistically significant. Conversely, 1,25(OH)_2_D_3_ significantly increased VDR expression in the presence of standard *Pg*LPS in a dose‐dependent manner. Similar tendencies were observed after co‐stimulation with 1,25(OH)_2_D_3_ and Pam3CSK4, but these effects were not significant. Treatment with IFN‐γ resulted in decreased VDR expression, which was independent of co‐stimulation with vitamin D_3_ metabolites. However, this reduction was also not statistically significant.

The influence of standard *Pg*LPS (1 µg/mL; Figure 6D), Pam3CSK4 (1 µg/mL; Figure 6E) or IFN‐γ (0.1 µg/mL; Figure 6F) on the 25(OH)D_3_‐triggered (0 to 100 nM) VDR gene expression levels in hPDLCs is demonstrated in Figure 6. Treatment with 25(OH)D_3_ minimally decreased VDR gene expression levels under physiological conditions, which was not statistically significant. The same tendency was observed during combined stimulation of 25(OH)D_3_ with standard *Pg*LPS. Pam3CSK4‐treated hPDLCs showed a slight increase of VDR gene expression levels due to stimulation with 25(OH)D_3_, but this effect was also not significant. Similar to 1,25(OH)_2_D_3_, 25(OH)D_3_ treatment with two different concentrations did not affect the IFN‐γ‐induced VDR gene expression.

Gene expression levels of CYP27B1 resulting from treatment of hPDLCs with 1,25(OH)_2_D_3_ (0 to 10 nM) or 25(OH)D_3_ (0 to 100 nM) in the presence and absence of either standard *Pg*LPS (1 µg/mL) or Pam3CSK4 (1 µg/mL) are presented in supplementary Figure S2 in online *Journal of Periodontology*. Inflammatory conditions had no effect on the basal or vitamin D_3_‐induced CYP27B1 expression in hPDLCs.

### 3.5 Protein concentration of osteocalcin and osteopontin in conditioned media of hPDLCs treated with vitamin D_3_ metabolites under physiological and inflammatory conditions

Osteocalcin and osteopontin protein concentration in supernatants of hPDLCs stimulated with 0 to 10 nM 1,25(OH)_2_D_3_ and 0 to 100 nM 25(OH)D_3_ in the presence and absence of standard *Pg*LPS (1 µg/mL), Pam3CSK4 (1 µg/mL) or IFN‐γ (0.1 µg/mL) were analyzed by ELISAs with a sensitivity of 156.5pg/mL and 31.25pg/mL, respectively, but could not be detected (data not shown). Notably, our experiments were performed in serum‐free DMEM, since media supplemented with 10% FBS contain high amounts of osteocalcin, which is over the range of commercial ELISA kits (10 ng/mL).

## 4 DISCUSSION

The aim of this study was to assess the bioactivity of 1,25(OH)_2_D_3_ and 25(OH)D_3_ in hPDLCs under inflammatory conditions, which were simulated by *Pg*LPS (standard and ultrapure), Pam3CSK4 or IFN‐γ. Analysis of VDR‐regulated genes osteocalcin and osteopontin, as well as VDR and CYP27B1 was performed via qPCR.

The bone gamma‐carboxyglutamic acid‐containing protein osteocalcin is encoded by the BGLAP gene, which is directly transcriptionally regulated by VDR.^33^ Osteocalcin is highly expressed during differentiation of osteoblasts.^34^ Likewise, the production of osteopontin, a sialic acid‐rich glycosylated phosphoprotein, is regulated by 1,25(OH)_2_D_3_ and strongly promoted when bone matrix is formed.^35^ Similarly to bone marrow‐derived MSCs, these two osteogenic markers have been shown to be enhanced by 1,25(OH)_2_D_3_ in hPDLCs.^24^, ^36^ The present in vitro study is the first to evaluate these effects in hPDLCs while simulating inflammatory conditions. On the one hand, such conditions were attained by stimulating the cells with TLR agonists, which have been shown to strongly enhance the inflammatory response of hPDLCs.^27^ On the other hand, inflammation was simulated by treatment with IFN‐γ, which is known to increase the immunomodulatory ability of hPDLCs and plays a substantial role in bone loss by mediation of the Th1‐type response.^37^, ^38^

Evaluating treatment with vitamin D_3_ metabolites under physiological conditions, our results show that not only 1,25(OH)_2_D_3_, but surprisingly also 25(OH)D_3_ significantly enhances the gene expression of osteocalcin by hPDLCs (Figures. 1 and 2). The effects of the vitamin D_3_ metabolites on osteopontin gene expression levels were clearly less pronounced (Figures. 3 and 4). Interestingly, such strong effects of 25(OH)D_3_ on osteocalcin and osteopontin gene expression have so far only been reported in bone‐marrow derived MSCs starting from a concentration five times higher than in the present study.^39^ In contrast, in our study this effect was observed at 25(OH)D_3_ concentrations similar to serum levels, which are physiologically relevant. This suggests that the alteration of systemic vitamin D_3_ levels might influence local periodontal homeostasis.

Transcriptional activation of VDR‐regulated genes induced by vitamin D_3_ metabolites was drastically affected by proinflammatory stimuli. The most pronounced effect was observed upon Pam3CSK4 treatment, which significantly diminished the expression of osteocalcin and osteopontin induced by 1,25(OH)_2_D_3_ and the expression of osteocalcin induced by 25(OH)D_3_ (Figures. 1 through 4). Pam3CSK4 is a synthetic TLR‐2 agonist, which elicits an even stronger inflammatory response of periodontal cells than TLR‐4 agonists.^27^ As shown in TLR‐2 deficient mice, activation of TLR‐2 is furthermore crucial in mediating bone loss in periodontitis.^40^ Our data showed that activation of NF‐κB might regulate the activity of VDR in hPDLCs. This effect might be due to the inhibition of Wnt‐ and bone morphogenetic protein‐signaling pathways.^41^ Alternatively, this could be due to the inhibitory effect of inflammatory stimuli on the retinoid x receptor, which facilitates VDR activation by 1,25(OH)_2_D_3_.^42^, ^43^ Interestingly, VDR is known to inhibit NF‐κB transcriptional activation and cytokine production in periodontal ligament cells.^25^, ^44^ Thus it seems that VDR and NF‐κB inhibit their activation in a reciprocal way. Such interaction of these transcription factors might play an important role in different inflammatory diseases and particularly periodontitis.

The effect of standard *Pg*LPS on the expression of VDR regulated genes was qualitatively similar to those of Pam3CSK4, but quantitatively less pronounced. These differences might be explained by the fact that standard *Pg*LPS is a less potent activator of NF‐κB response in hPDLCs than Pam3CSK4.^27^, ^32^, ^45^ Furthermore, ultrapure *Pg*LPS had no significant effect on vitamin D_3_ induced response in hPDLCs (see Figure S1 in online *Journal of Periodontology*). A previous study shows that standard *P. gingivalis* LPS activates both TLR‐2 and TLR‐4, whereas ultrapure *Pg*LPS acts exclusively through TLR‐4. Moreover, standard *Pg*LPS induces markedly stronger inflammatory response in monocytes and hPDLCs (Behm et al., not yet published).^46^ Thus, inhibition of the vitamin D_3_‐induced response seems to depend on the degree of NF‐κB activation. This assumption is supported by our findings that the Pam3CSK4‐ and standard *Pg*LPS‐induced decrease of the vitamin D_3_‐triggered response in hPDLCs is recovered under NF‐κB inhibition. Furthermore, it could also explain the observation that the response to vitamin D_3_ was not inhibited by IFN‐γ, which is a rather weak activator of NF‐κB signaling.^47^

We further found that the gene expression levels of VDR were not affected by 1,25(OH)_2_D_3_ and 25(OH)D_3_ (Figure 6), which is in contradiction with the study of Tang et al. who found a 3‐fold higher VDR expression after treatment with 10 nM 1,25(OH)_2_D_3_.^48^ This discrepancy could be explained by the fact that Tang and colleague used osteogenic induction medium supplemented with 10‐mM β‐glycerophosphate, 50‐µg/mL ascorbic acid, 10^−7^ M dexamethasone, and 20% FBS and treated the cells for 6 days.^48^ These artificial additives of osteogenic medium might influence the responsiveness to vitamin D_3_, but this is a question that needs to be further explored. TLR agonists standard *Pg*LPS and Pam3CSK4 had no influence on the gene expression levels of VDR (Figure 6). This observation is in accordance with the findings of Nebel et al., who treated hPDLCs with *Escherichia coli* LPS.^24^ However, 1,25(OH)_2_D_3_ significantly enhanced VDR expression in the presence of standard *Pg*LPS (Figure 6). Qualitatively similar effects have been demonstrated by Pramanik et al. in human monocytic THP‐1 cells after combined treatment with 1,25(OH)_2_D_3_ and lipopolysaccharide.^49^ This suggests some potential interactions between TLR and VDR responses, which should be further investigated.

The inhibitory effects of Pam3CSK4 and standard *Pg*LPS on the gene expression of VDR‐regulated genes seem to be independent of the expression of proteins involved in vitamin D metabolism. Inhibition of VDR regulated genes was not accompanied by the decrease of VDR gene expression levels in hPDLCs. Therefore, the effects of Pam3CSK4 and standard *Pg*LPS are associated with inhibition of VDR transcriptional activity. As shown in supplementary Figure S2 in online *Journal of Periodontology*, gene expression levels of CYP27B1, which is involved in local conversion of 25(OH)D_3_ into 1,25(OH)_2_D_3_ in hPDLCs, are not affected by Pam3CSK4 or standard *Pg*LPS. This finding is underlined by the fact that both TLR agonists diminished the response to 1,25(OH)_2_D_3_ and 25(OH)D_3_ by a similar extent. The effect of IFN‐γ on the expression of VDR regulated genes was less obvious than that of the TLR agonists as can be seen in Figures 1 through 4. For example, IFN‐γ led to a slightly increased osteocalcin expression induced by 10 nM 1,25(OH)_2_D_3_, but this effect was not significant. In contrast, osteocalcin expression triggered by 25(OH)D_3_ was significantly diminished by IFN‐γ. The different effects of IFN‐γ on the 1,25(OH)_2_D_3_ and 25(OH)D_3_‐induced response imply that this cytokine influences the bio‐activation of 25(OH)D_3_ by hPDLCs. This assumption is supported by previous data showing that CYP27B1 is regulated by IFN‐γ in macrophages, vascular endothelial cells, and keratinocytes.^50^ The possibility of such a regulation in hPDLCs, as well as its potential physiological importance needs to be elucidated by future studies.

In our experiments, neither osteocalcin nor osteopontin could be detected in the supernatants by ELISA, which could be explained by several factors. First of all, our study was conducted with primary undifferentiated cells to more closely resemble the in vivo situation and therefore may result in low protein contents that are not detectable by ELISA (lower detection limit: 156.5 pg/mL). Furthermore, our treatment time was rather short compared with other studies, which could contribute to the low protein level. Lastly, our treatment with vitamin D_3_ was done in the absence of different additives like serum, dexamethasone, and ascorbic acid, which are usually present in osteogenic medium, but are rather artificial and non‐relevant physiologically. According to our measurements, medium supplemented with 10% serum contains more than 10 ng/mL osteocalcin, which impedes analysis of this protein with ELISA. Our experimental protocol facilitates to discriminate between the effects of vitamin D_3_ metabolites and avoids the influence of other artificial factors, but does not allow detection of protein production. Nevertheless, our data clearly suggest that transcriptional activation of vitamin D_3_ regulated genes is clearly affected by inflammatory conditions.

Several reviews and original articles about the relationship between vitamin D_3_ deficiency and periodontal disease have been conducted so far. However, most of the data were collected in observational cross‐sectional studies and casual associations between vitamin D_3_ deficiency and periodontitis remain unclear.^15^ In particular, there is no clear evidence for beneficial effects of vitamin D_3_ supplementation during periodontal therapy, especially in the initial phase. Such observations are rather surprising, considering the potentially positive impact of vitamin D_3_ on maintaining periodontal health, such as induction of antibacterial mechanisms, strengthening of the physical barrier, inhibition of inflammatory processes, and support of wound healing.^15^, ^34^ Thus, our study may provide an explanation for the lacking benefit of vitamin D_3_ during initial periodontitis treatment, in which inflammation is markedly pronounced.

## 5 CONCLUSIONS

Summarizing the results of the present study indicates that the bioactivity of vitamin D_3_ might be diminished in periodontal tissue of periodontitis patients, which might be due to disruption of the vitamin D_3_ metabolism. Therefore, it may be possible that the beneficial effects of vitamin D_3_ supplementation are dampened in those individuals. Revealing the underlying mechanisms of diminished vitamin D_3_ bioactivity during inflammation could be a future goal to enhance the effectiveness of vitamin D_3_ supplementation as adjunctive periodontal therapy.

## AUTHOR CONTRIBUTIONS

O.A. and A.B. were responsible for conzeptualization. O.A., A.B. and B.K. were involved in methodology. Validation was performed by C.B. and J.G. The formal analysis was conducted by A.B. B.K. and A.B. were responsible for investigations. The resources have been provided by A.M. and X.R. J.G. was responsible for data curation. Writing of the original draft was performed by A.B. and O.A, followed by review and editing by C.B., A.B. and X.R. A.B. was responsible for visualization. Supervision and project administration was conducted by O.A. Funding acquisition was performed by O.A., X.R. and A.B.

## Supporting information

Supplementary Information

## Footnotes

* Sigma‐Aldrich, St. Louis, MO.

† Gibco, Carlsbad, CA.

‡ Gibco, Carlsbad, CA.

§ eBiosciences, San Diego, CA.

¶ eBiosciences, San Diego, CA.

# Cayman Chemical, Ann Arbor, MI.

∥ Cayman Chemical, Ann Arbor, MI.

** Invivogen, San Diego, CA.

†† Invivogen, San Diego, CA.

‡‡ PeproTech, Rocky Hill, CT.

§§ Sigma‐Aldrich, St. Louis, MO.

¶¶ Merck Millipore, Burlington, VT.

## TaqMan Gene Expression Cells‐to‐CT‐kit, Applied Biosystems, Foster City, CA.

∥∥ Primus 96 advanced thermocycler, PeqLab/VWR, Darmstadt, Germany.

*** TaqMan Gene Expression Assays, Applied Biosystems, Foster City, CA.

††† ABI StepOnePlus, Applied Biosystems, Foster City, CA.

‡‡‡ DuoSet ELISA kits, R&D Systems, Minneapolis, MN.

§§§ IBM, Armonk, NY.
