# Supplementary material for: Transcriptional activity of vitamin D receptor in human periodontal ligament cells is diminished under inflammatory conditions
Source: J Periodontol. 2020 Jun 21;92(1):137–48. doi: 10.1002/JPER.19-0541 (PMC7891446; doi:10.1002/JPER.19-0541)
Supplement: Supplementary file 1 — Supplementary Information [file JPER-92-137-s001.docx]

**Supplementary data**

**
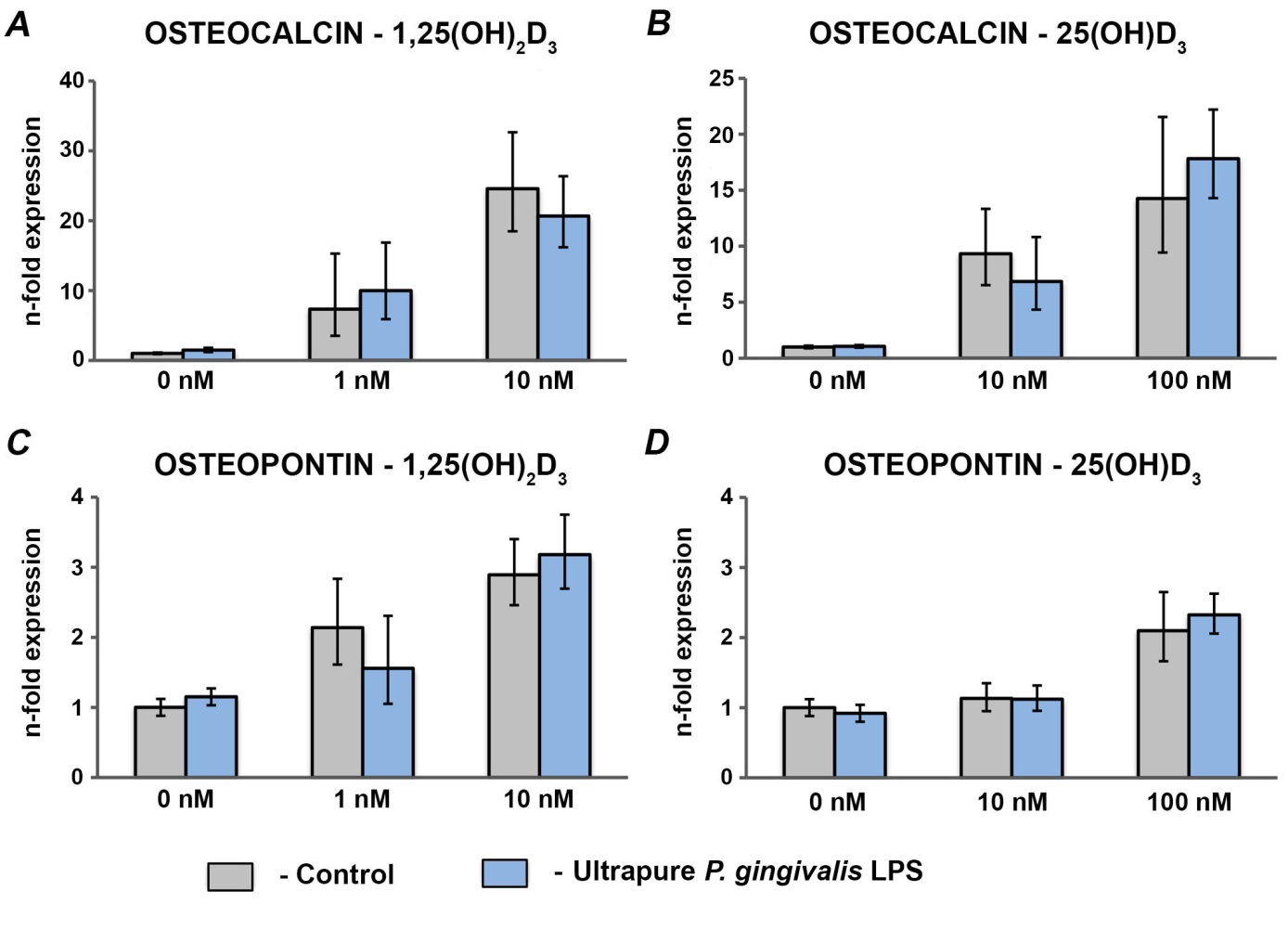
**

**Figure S1. Gene expression levels of osteocalcin and osteopontin in hPDLCs treated with 1,25(OH)2D3 or 25(OH)D3 under physiological and inflammatory conditions and NFκB inhibition**

hPDLCs of six healthy donors were treated with 1,25(OH)_2_D_3_ (1-10nM) or 25(OH)D_3_ (10-100nM) in the presence and absence of ultrapure *P. gingivalis* LPS (1µg/ml) + sCD14 (0.2µg/ml) for 48 h. Osteocalcin (A, B) and osteopontin (C, D) gene expression levels were measured with qPCR. Y-axes show the n-fold expression of osteocalcin and osteopontin expression, respectively, compared to untreated cells (=1). GAPDH served as endogenous control. Data are presented as mean ± S.E.M. of 6 different donors. *signficant difference between groups, p<0.05


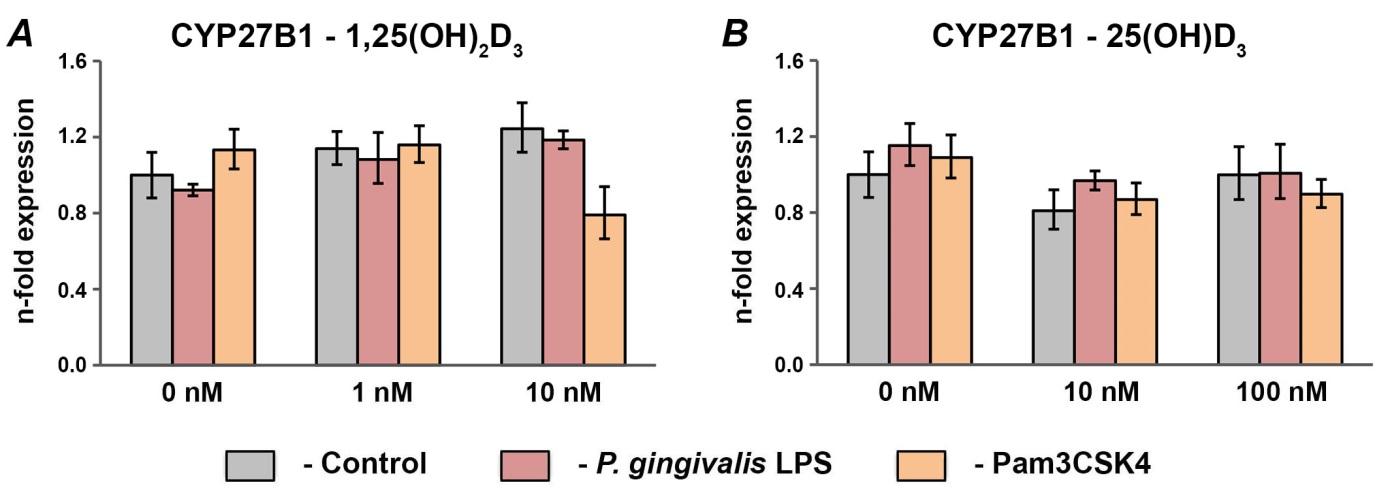


**Figure S2. Gene expression of CYP27B1 in hPDLCs treated with 1,25(OH)_2_D_3_ or 25(OH)D_3_ under physiological and inflammatory conditions**

hPDLCs of six healthy donors were treated with 1,25(OH)_2_D_3_ (0-10nM) or 25(OH)D_3_ (10nM, 100nM) in the presence and absence of standard *P. gingivalis* LPS (1µg/ml) + sCD14 (0.2µg/ml) or Pam3CSK4 (1µg/ml) for 48 h. CYP27B1 gene expression levels were measured with qPCR. Y-axes show the n-fold expression of CYP27B1 expression compared to untreated cells (=1). GAPDH served as endogenous control. Data are presented as mean ± S.E.M. of 6 different donors.
